# Double-Diamond Model-Based Orientation Guidance in Wearable Human–Machine Navigation Systems for Blind and Visually Impaired People

**DOI:** 10.3390/s19214670

**Published:** 2019-10-28

**Authors:** Xiaochen Zhang, Hui Zhang, Linyue Zhang, Yi Zhu, Fei Hu

**Affiliations:** 1 Department of Industrial Design, Guangdong University of Technology, Guangzhou 510006, China; xzhang@gdut.edu.cn (X.Z.); or; 2 School of Communication and Design, Sun Yat-Sen University, Guangzhou 510275, China; 3 School of Industrial Design, Georgia Institute of Technology, Atlanta, GA 30332, USA

**Keywords:** tactile feedback, navigation aids, user-centric design, double diamond, blind and visually impaired people

## Abstract

This paper presents the analysis and design of a new, wearable orientation guidance device in modern travel aid systems for blind and visually impaired people. The four-stage double-diamond design model was applied in the design process to achieve human-centric innovation and to ensure technical feasibility and economic viability. Consequently, a sliding tactile feedback wristband was designed and prototyped. Furthermore, a Bezier curve-based adaptive path planner is proposed to guarantee collision-free planned motion. Proof-of-concept experiments on both virtual and real-world scenarios are conducted. The evaluation results confirmed the efficiency and feasibility of the design and imply the design’s remarkable potential in spatial perception rehabilitation.

## 1. Introduction

The World Health Organization factsheet stated that there are approximately 217 million people suffering from moderate to severe visual impairment, with 36 million blind people worldwide [1,2]. Compared to normally sighted people, they have difficulties in accessing visual cues in daily surroundings.

Obviously, blind and visually impaired (BVI) people have a strong dependency on travel aids, tools, and advanced systems [3]. Fortunately, advances in robotics, computer vision, geographic information systems (GISs), and multimodal sensory technology allow for modern smart systems to perform mapping, positioning, and decision-making while users navigate through urban areas [4].

Conventional travel aids for BVI, including white canes, guide dogs, and volunteers, have their intrinsic restrictions [5]. Modern assistive solutions borrow power from mobile computing, robotics, and autonomous technology to overcome these restrictions, as well as to empower new capabilities such as representing the external world digitally in hybrid human–machine systems [5,6].

According to References [7,8,9], numerous systems are capable of understanding the features of surroundings efficiently and effectively by taking advantage of various onboard sensors and intelligence decision-making advances, especially for travel scenarios. However, most of the studies treated the human–machine system as a conventional mobile robotics system, e.g., using waypoint-based path planning and discrete instructions [5]. The lack of focus on human-centric issues in innovations brings about an insurmountable gap between human and machine cognition [6].

Specifically, BVI people prefer continuous guidance to discrete guidance, so that they retain the tracking of steering instructions at will. They prefer smooth guidance to interruptive coded instructions, so that they do not have to pause to understand the guidance before proceeding. To enhance the readability of this paper, we use the term “continuous” to indicate that the instructions are continuously readable, with clear one-to-one correspondence with the corresponding physical meaning at any time, the term “smooth” to indicate that the reading and perception of guidance instructions are not interruptive and are burden-less, so that people do not have to suspend their current action to understand the guidance instructions.

Moreover, the capability of guidance following is also a part of human spatial cognition. A series of studies by Mosers, winner of the 2014 Nobel Prize in medicine, found that the ability of a moving person to roam in space depends on how much they know about the scene [10]. By consciously imagining and perceiving spatial position and orientation, BVI people may obtain a certain degree of situation awareness and spatial cognition in navigation [11]. Successful design and application of an orientation guidance system may potentially aid training programs for spatial perception rehabilitation.

In this work, we propose a new design of a sliding tactile feedback wristband that guides orientation for BVI navigation systems. The design process is based on the double-diamond design model, with a clear, comprehensive, and visual description of the design thinking process. After the four-phase design process, a design aiming to resolve the numerous limitations of current state-of-the-art solutions is proposed. A Bezier curve-based path planning is proposed to guarantee collision-free path planning. The proof-of-concept prototype is evaluated with blindfolded and BVI volunteers. Proof-of-concept experiments on both virtual and real-world scenarios are conducted. The evaluation results confirm the efficiency and feasibility of the design and imply its potential in applications of spatial perception rehabilitation.

The contributions of this work are manifold. Firstly, it is the first hand-free tool that provides “smooth” and “continuous” sliding tactile orientation guidance for BVI pedestrians. Secondly, it successfully applies the double-diamond design model to address the numerous limitations of existing acoustic and vibration orientation guidance solutions. Thirdly, the experiments in a virtual test field and real test field imply the potential of the designed wristband and testbed in spatial perception rehabilitation applications. Fourthly, a proof-of-concept prototype was produced, which was demonstrated to be functionally capable, portable, and affordable.

The rest of the paper is organized as follows: Section 2 reviews relevant related works. Section 3 presents the details of the design using a double-diamond design model. Section 4 presents the experiments and results. Section 5 presents the discussion regarding the design and experiment. Section 6 summarizes the work.

## 2. Related Works

### 2.1. Smart Travel Aids for BVI People

Recent advances in sensor technology and artificial intelligence supported the design and development of smart travel aids for BVI people. Katz [12] designed an assistive device that aids BVI people in multi-scale object identification and route planning. Zhang [13] proposed a hybrid-assistive system using both stereo sensors and a web camera to localize BVI users on pre-built maps. Ahmetovic [14] designed a waypoint-based BVI navigation system that takes advantage of previously deployed wireless beacons in positioning. Remarkably, Bing [15] used a sparse-mapping-based Project Tango Tablet with no extra sensor to guide BVI users. Zhu [16] proposed and implemented the ASSIST (Assistive Sensor Solution for Independent and Safe Travel) system on a Project Tango smartphone that helps BVI users navigate indoors. To date, many researchers and scientists made efforts to deliver affordable high-tech travel aids for BVI users [17]. Benefiting from the rapid development of artificial intelligence and autonomous driving [18], the localization, mapping, and navigation technology for smart travel aids are developing rapidly and promisingly day by day. Nevertheless, most of the existing devices were only tested in laboratory scenarios [19,20,21,22]. One of the core reasons is that the current human–machine interaction-based cooperative perceptions are asynchronized and insufficient. In other words, humans and machines each have their own perception in tasks. If humans and machines could simultaneously share and understand their perceptions, the difficulty in applying human–machine system in the real world would be limited. However, the fact is that humans take time and effort to understand real-time machine perception, while machines can only feed a limited portion of their perceptions to humans.

**Figure 1:**
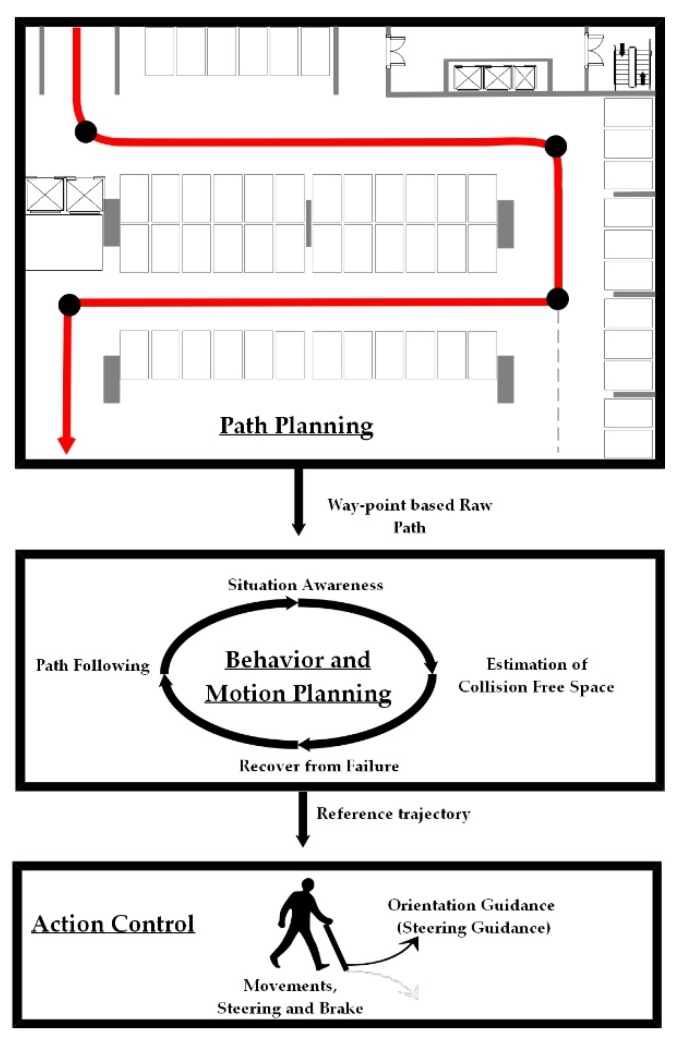
A three-layer planning-related decision-making structure for blind and visually impaired (BVI) navigation systems.

Enlighted by the hierarchy of decision-making processes in self-driving urban vehicles [23], we considered planning-related decision-making of BVI navigation systems in three layers, as shown in Figure 1. The top layer plans the waypoint-based raw path for BVI pedestrian, a procedure similar to autonomous mobile robots. The middle layer generates behavior and motion plans assuming that the reference trajectory can be carried out by BVI users. The bottom layer aims to guarantee that the reference trajectory can be followed or an equivalent goal can be reached via human–machine interactions. Our work contributes to the bottom action control layer.

### 2.2. Human–Machine Interaction and Multimodal Feedback for Orientation Guidance

Most BVI travel aids use auditory and tactile interactions in human–machine systems [22]. Shah [24] used a handheld array of actuators to convey complex meaning via various vibrations. Rizvi [25] presented a simple navigation aid for BVI people using a buzzer and haptic feedback to deliver obstacle alerts. Sohl-Dickstein [26] used stereo sound to provide spatial information. Meanwhile, Bai [27] proposed a lightweight glasses-mountable device to aid BVI people using guiding sounds. Patil [28] designed NavGuide, an electronic aid using vibration and audio to provide simplified high-priority surrounding information. Amemiya [29] proposed a pseudo-attraction haptic direction indicator, but the device was handheld and bulky. Kaushalya developed “AKSHI” system [30], which verbally guides the BVI user to avoid obstacles. Cardillo [31] used vibration and acoustic signals to indicate the objects of interest in navigation. Although significant progress took place, there is still a nonnegligible deficiency in feedback mechanisms in the action control layer. There are two major issues. Firstly, the capabilities of fulfilling descriptive guidance instructions differ from BVI user to user. In practice, they cannot acquire sufficient guidance from discrete instructions to adjust their actions in each action period, e.g., steering and movements. Secondly, it is unsatisfactory for BVI users to spend much time and effort on perceiving the instructions, especially for coded guidance instructions. Most state-of-art works are insufficient in one of the above two issues in the action layer, while they pay more attention to the path-planning, behavior, and motion-planning layers. Therefore, a continuous and smooth action control layer guidance mechanism is highly worthwhile.

### 2.3. Design Thinking in Human-Centric Innovations

Design thinking is a methodology to achieve human-centric innovation while ensuring technical feasibility and economic viability [32,33]. Instead of emphasizing objective technic-centric evaluation criteria, design thinking treats the innovative design as a comprehensive and iterative human-centric problem-solving process. Throughout the process, several different design science approaches are proposed and applied to explore and fill the gap between human needs and implementations. In addition to conventional stakeholder analysis [34], competitor analysis [35,36], the British Design Council [37] proposed the double-diamond design model consisting of problem and solution spaces. This model allows for a systematic design analysis with double diverge–converge phases to find a feasible solution. The attempted “continuous” and “smooth” solutions of this work are coincident with Nielsen usability heuristics [38]. Specifically, “continuous” denotes that the system should always keep users informed about the status of guidance following, i.e., the principle of “visibility of system status”. It also allows a timely adjustment of orientation, i.e., the principle of “error prevention”. On the other hand, “smooth” denotes that the instructions do not take much time to learn, read, and understand, i.e., the principle of “recognition rather than recall” and “flexibility and efficiency of use”. 

## 3. Design of BVI Orientation Guidance Using a Double-Diamond Design Model

**Figure 2:**
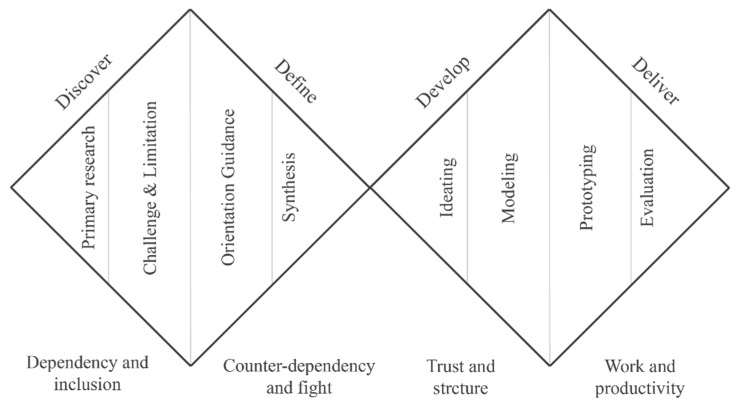
The four-stage double-diamond design model.

The double-diamond design model [37] is considered among the most efficient and convincing design thinking process models since being proposed by the British Design Organization in 2005. The model entails emphasis on problem analysis as a basis for creating solutions for clients, and it presents four main stages across two adjacent diamonds. The two diamonds are the problem and solution spaces. In each space, a diverging phase that expends the space is followed by a converging phase that narrows the space, as shown in Figure 2. The model distinguishes itself from others by systematically splitting the human-centric design into two successive diverging–converging phases. To synthesize the design opportunity and contact points gathered from the need-finding stage, insights and primary design goals are formulated in the following converging stage. Then, the collection of user and expert feedback after ideating and modeling in the idea implementation stage allows converging to a final solution in the finalization stage [33]. The process is highly couplable with user-centric usability design principles such as visibility of system status, match between system and the real world, user control and freedom, flexibility, and efficiency of use [39]. Moreover, the four stages are termed discovery, define, develop, and deliver [37]. 

### 3.1. Discover Stage

#### 3.1.1. Primary Research Goal

There is a consensus that BVI users’ quality of life would be significantly improved by smart travel aids that take advantage of scientific advances and service innovations [4]. In this work, we consider human–machine navigation systems which are capable of performing localization, path and motion planning, and motion action guidance. Currently, technology for localization and planning is significantly improved due to the rapid development of automatous robots and self-driving vehicles [40]; however, the progress of autonomous systems is by no means an equivalent bonus for BVI human–machine systems since there is a lack of adequate human–machine cooperative perception support. Among the numerous human–machine cooperative perception issues in BVI travel aids, orientation guidance is one of the most important since it determines whether the motions planned by the machine can be properly carried out by humans in real time.

Researchers [15,41,42] reported the significance of acquiring augmented localization information by perceiving and processing the surrounding spatial information via multimodal perception in BVI navigation. Joseph [43] stated that the acquired augmented localization information significantly supports the motion planning and navigation of autonomous robots and commercial self-driving vehicles. However, these achievements are still in the machine perception stage instead of synchronized human–machine cognition. Consequently, BVI human–machine systems are theoretically useful but only partially meet the requirements of application; they lack a suitable bridge to convey the knowledge and decisions of machines to BVI people especially in the action controls. A better bridging tool or service that conveys navigation-related knowledge and guidance from the machine to the BVI user needs to be designed [3]. 

#### 3.1.2. Challenges and Limitations of Existing Work

According to Bujacz and Balan [44,45], acoustics is the most suitable channel to deliver long-term path-planning knowledge since the other candidate interaction channels may not be able to deliver much knowledge of rich data in a timely manner with a certain degree of ambiguity. However, when it comes to the planning details in the action control layer, there are different designs.

Most existing designs rely on acoustic feedback [27,46,47,48]. Well-formulated verbal instructions of motion cues are the most popular, while other studies either used frequency modulation-based three-dimensional (3D) acoustic signals or composed a mixed symphony (or noise) to guide BVI people.

Another widely accepted opinion is that tactile-based feedback is qualified to dominate the motion-related feedback in BVI navigation. For instance, vibration-based interactions are used to imply further motion actions and alerts for upcoming obstacles or objects of interests.

Moreover, lots of recent studies provided combinations of acoustic and tactile feedback for guidance in action control [15,22]. Their integration delivers relatively egocentric solutions that seek a balance between cognition loads and explicit instructions.

As a matter of fact, the existing solutions have their inherent limitations in action control planning. Firstly, designs relying on acoustic feedback suffer from the following problems:

*Inevitable information delivery latency*. The delivery and decoding of audio instructions are not instant. In most cases, the verbal commands are not suitable for reaction time-sensitive tasks such as motion and orientation guidance.

*Excessive learning burden*. The learning burden for verbal commands based on natural languages is not significant. However, when it comes to frequency-based sound instructions, the learning burden becomes immense.

*Difference between descriptive instructions and executive actions*. Acoustic instructions deliver descriptive instructions with a certain degree of ambiguity. This limitation results from the inherent low capacity of the linear acoustic transmission channel; complete semantics are composed only after a certain period of communication.

*None-multiplexing channel*. In most cases, it is not recommended that the channel is shared by more than one communication task. The interference between independent signals is serious and inevitable. Moreover, the length of communication time is proportional to the information capacity. Since hearing is the most important sensory receiver for BVI people in daily life, the non-multiplexing property makes the hearing channel more precious.

*Results-oriented instructions*. The instructions aim to accomplish short-term and medium-term goals, i.e., they are results-oriented, which makes guidance during a detailed action insufficient.

Meanwhile, the existing tactile feedback-based designs have the following limitations:

*Discrete guidance on continuous motion tasks*. Most of the solutions use three to five vibration units to indicate the approximate orientation. Combinations of vibrations allow the BVI user to find clues of motion guidance. Nonetheless, this is contradictory to the continuous nature of dynamics and motion in navigation.

*Instructions are prone to be misperceived*. Firstly, depending on the tactile perception of the specified user, the combination of vibrations might be perceived quite differently. Secondly, the partial misbehaviors of vibration units are difficult to be self-diagnosed during operation.

*Raw ergonomic user experience*. The frequent vibration makes navigation annoying. Moreover, it tends to shorten the device’s life span.

Despite fusing the merits of the acoustic and tactile mechanisms, the existing integrated designs have the following flaws:

*Waypoint finding-based routing and motion planning are not human-friendly*. The waypoint finding-based planning is perfect for autonomous robots, but it is not reasonable to expect that BVI users could follow the planned track as well as robots.

*Discrete guidance on continuous motion tasks*. Existing designs combining acoustic and tactile feedback do not resolve the problem of discrete guidance and, thus, guessing of BVI users plays an important role in navigation. Consequently, the sense of reliability and security of BVI users significantly suffers.

*Raw ergonomic user experience.* For action control, the acoustics are essential but difficult to follow. For better adoption of designs, the essential vibrations must be improved or replaced.

*Bulky*. Although the volume and complexity of sensory composition is not a scientific problem, it significantly influences the usability and the device’s potential adoption. According to References [3,40], a number of recent studies compensated for capability with bulk. This also led to higher prices and lower robustness in most cases, which prevents the future commercial versions from being widely adopted by price-sensitive BVI users.

### 3.2. Define Stage

After refining the research and design challenges, it was concluded that there is a grand demand of feasible action control mechanisms and guidance devices.

#### 3.2.1. Orientation Guidance in a BVI Human–Machine System

Following insights by Long [49,50], in this work, we define orientation as the knowledge of one’s direction relative to egocentric frames and the ability to keep track of the spatial relationships in motion. Orientation guidance is provided by a certain device or service through multimodal feedback or interaction. The goal is to allow BVI users to access orientation-related information or cues to support decisions and actions in real time. 

#### 3.2.2. Synthesis Design Opportunities

*Human body tactile sensitivity map*

**Figure 3:**
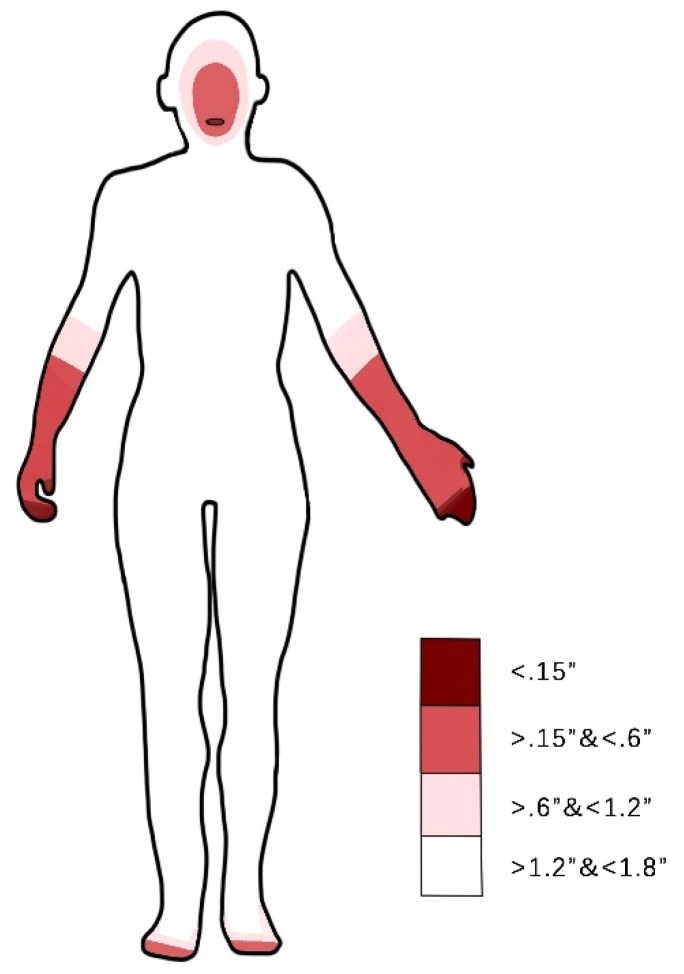
Average distance in two-point discrimination sensitivity test on body locations; data were taken from the associated website of Reference [51].

According to Zeagler [51], tactile feedback may be used to support BVI navigation. For orientation guidance at the action control level, tactile stimuli can be mapped onto an azimuth egocentric frame, which is, hence, a primary choice. As shown in Figure 3, a body tactile sensitivity map must be considered while choosing a wearable design (data were taken from Reference [51]). Specifically, the two-point discrimination sensitivity test finds the distance needed to distinguish two compass points simultaneously applied to the skin [52]. In most cases, the two-point discrimination map reflects the reaction sensitivity of the skin. 

*BVI human–machine systems*

For any orientation guidance module, the human–machine navigation system must have a supporting main system. By considering applicability and functionality, we chose the ARCore-based navigation system [53] as the main system providing extra sensors, power, and multimodal interactions.

*Spatial perception rehabilitation for BVI*

Schinazi and Majerova’s study [54,55] indicated that BVI people face difficulties in spatial perception and imagination, especially in performing dead reckoning in strange places. An advanced level of design seeks an orientation guidance that provides a convenient way to train and strengthen the spatial perception of BVI people.

### 3.3. Develop Stage

Usability and adoptability are the basis of product design. Usability design focuses on product functionality. Nielsen divided it into five evaluation dimensions: learnability, memorability, efficiency, fault tolerance, and user satisfaction [56]. Hartson analyzed usability from the two levels of usefulness and ease of use [57]. The international standard ISO-9241 (International Organization for Standardization-9241) defines it as “the effectiveness, efficiency, and satisfaction of a particular user in achieving a particular goal in a particular scenario” [58]. Like developments in industrial design, usability-oriented product design cannot fully fulfill demands; meanwhile, the multi-dimensional experience-oriented design is highly reputed. Experience design is especially concerned with the subjective feelings that products bring to users. Tom Wood believes that experience design is a design practice that focuses on both process and outcome, specifically the relevance of experience to user needs and context. 

In navigation, orientation guidance cooperates with cognitive modules such as sensory distance directors, computer visual cognition modules, anchor-based re-localization modules, and radio frequency positioning modules to guide movements. As an essential component of the cooperative cognition in human–machine systems, orientation guidance is supposed to be concisely developed under specified conditions that fit the demands of BVI navigation.

According to the tactile sensitivity body map in Figure 3, designs are chosen to be attached to the hand or forearm [29], foot [59], or tongue [60]. Moreover, the design improves the limitations of existing studies presented above.

Tactile feedback is delivered in real time to guide the BVI user, which means that the perception of feedback should be as simple as possible to ease the burden of perceiving feedback. A polar steering style is preferred since it can be projected onto egocentric azimuth orientation maps. The sliding tactile feedback is chosen to instruct upcoming motion directions continuously. Meanwhile, one specific direction is clearly presented at any given time. This also guarantees clear and reliable orientation cues. Making the instructions simple and matching human natural instincts and habits are important to lessen the learning burden and the possibility of misunderstanding. More importantly, the straightforward steering style makes the navigation pleasant instead of annoying. Since the guidance is no longer discrete, the path planning is free from waypoint finding. This simple yet efficient feedback style also allows the design to be inexpensive and portable.

**Figure 4:**
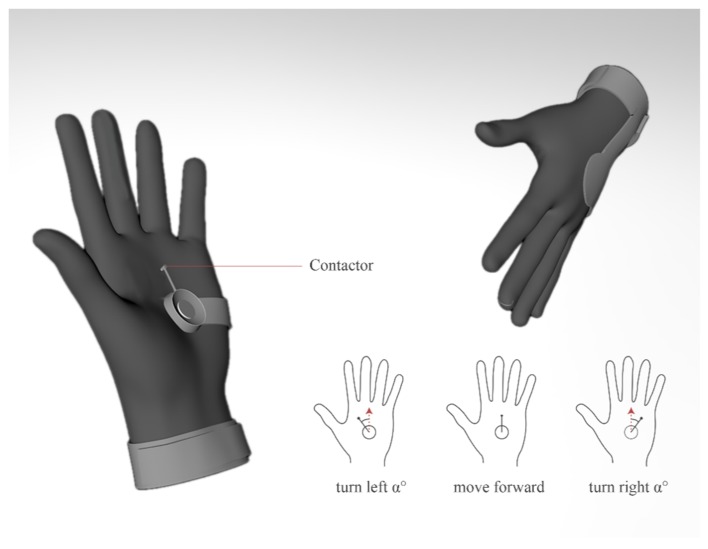
The design concept of the guidance glove.

Based on the above conceptual ideation, two independent designs were derived. To avoid distraction, the non-essential steps and procedures are described only briefly. The first design is a guidance glove that works on palms and is sensitive to pressure, as shown in Figure 4. The sliding pressure contactor driven by a steering gear is applied to indicate orientation guidance. A coordination projection between the palm and egocentric frame is applied, whereby a scaled-down virtual user standing on the palm of a left hand is facing toward the middle finger of the palm. The real-time contactor’s pointing direction indicates the suggested direction for forthcoming movements.

**Figure 5:**
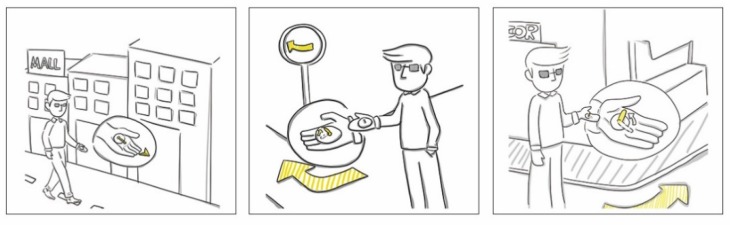
Typical application scenarios of guidance glove.

Typical application scenarios of the guidance glove are shown in Figure 5. The cognitive translation from tactile feedback to egocentric spatial perception is intuitive, which alleviates the burdens associated with learning and perception.

**Figure 6:**
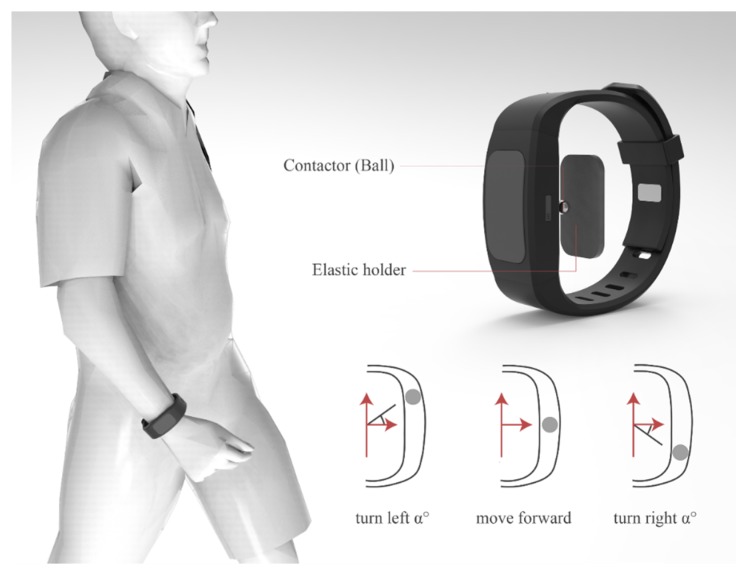
The design concept of the guidance wristband.

The second design is a guidance wristband that uses a steering-style pressure contactor on the wrists to indicate the instructed motion direction to BVI users, as shown in Figure 6. A coordination projection between the wrist frame and egocentric frame is applied, whereby a scaled-down virtual user is standing on the wrist of a left hand, and the egocentric frame and the wristband frame are coincident. The real-time contactor’s pointing direction indicates the suggested direction for upcoming movements.

### 3.4. Deliver Stage

#### 3.4.1. Prototyping

**Figure 7:**
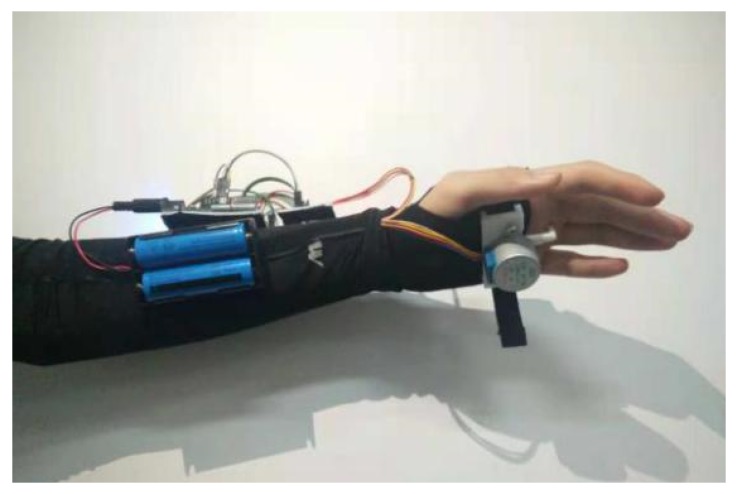
Proof of concept prototype of guidance glove [53].

The guidance glove (Figure 7) was prototyped on an Arduino Uno with a 28BYJ-48 step motor manufactured by (Quason, sourced from Shenzhen, China.) A Dofly manufactured HC-05 MCU (Microcontroller Unit) compatible Bluetooth module was adapted for communication, and the main body supporting the structure was three-dimensionally (3D) printed by UPBOX+. The mentioned mechanical and communication components are available from major e-commerce website like Amazon and Alibaba, in most regions in America, Europe and Asia.

This prototype was tested with four blindfolded and four BVI volunteers; part of the development and testing was conducted in our previous study [53]. Compared with conventional vibration feedback, the guidance glove guides the user smoothly, and the sensing experience is natural and pleasant. However, according to the subjects, some features heavily impair the experience. Firstly, the device occupies the palm, which is essential for conducting daily activities. Secondly, the palm is not always flat, which results in inadequate contact between the contactor and the palm.

**Figure 8:**
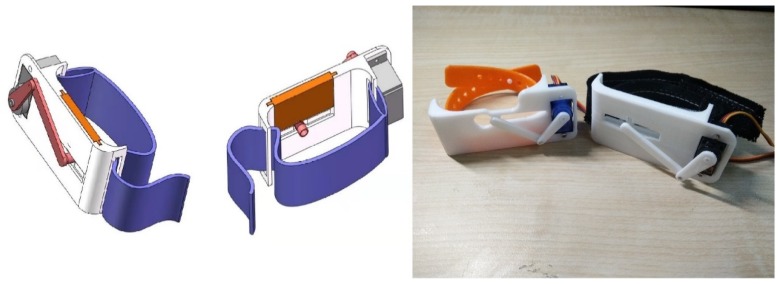
Proof-of-concept prototype of guidance wristband.

The guidance wristband (Figure 8) was prototyped on an Arduino Uno with either an SG90 standard steering motor or an AFRC D1015 linear steering motor. The main body was 3D printed by UPBOX+ with Poly-flex TPU95 elastic material. The mentioned mechanical and communication components are available from major e-commerce website like Amazon and Alibaba, in most regions in America, Europe and Asia. The testing and evaluation of the design are illustrated in the experimental section.

#### 3.4.2. Bezier-Curve Based Planning

Continuous orientation guidance requires motion planning unlike that used in conventional waypoint finding mechanisms. Instead of seeking the shortest or most inexpensive solution, the planning pursues smooth and collision-free motions. The smoothness is generally expressed in terms of continuity [61], i.e., geometric continuity or parametric continuity. In this work, the smoothness is expressed by geometric continuity. To achieve geometric continuity of motion, we used a Bezier curve-based algorithm [62] to plan the guidance. A Bezier curve of degree n is defined as

(1) $$P(t)=\sum_{i=0}^{n}B_{i}^{n}(t)P_{i},$$

where $P_{i}$ denotes the control points, and $B_{i}^{n}(t)$ is a Bernstein polynomial.

(2) $$B_{i}^{n}(t)=\left(\begin{array}{c} n\\ i \end{array}\right){\left(\frac{t_{1}-t}{t_{1}-t_{0}}\right)}^{n-i}{\left(\frac{t-t_{0}}{t_{1}-t_{0}}\right)}^{i}\;\;\;\;i\in \{0,1,\ldots ,n\}.$$

The paths generated by the Bezier curve have the following merits:

They always pass through the starting point $P_{0}$ and ending point $P_{k}$.

They are always tangent to the lines connecting consequential control point pairs.

They form curves to avoid the collision area surrounded by control points.

They form straight lines if there is no collision area.

The orientation guidance module aims to guide the BVI user according to the planned navigation path. In other words, given a predefined path, it makes efforts to generate sub-level path planning to follow the predefined path. In simpler terms, it guides the BVI user toward selected far-ahead points on the path.

**Figure 9:**
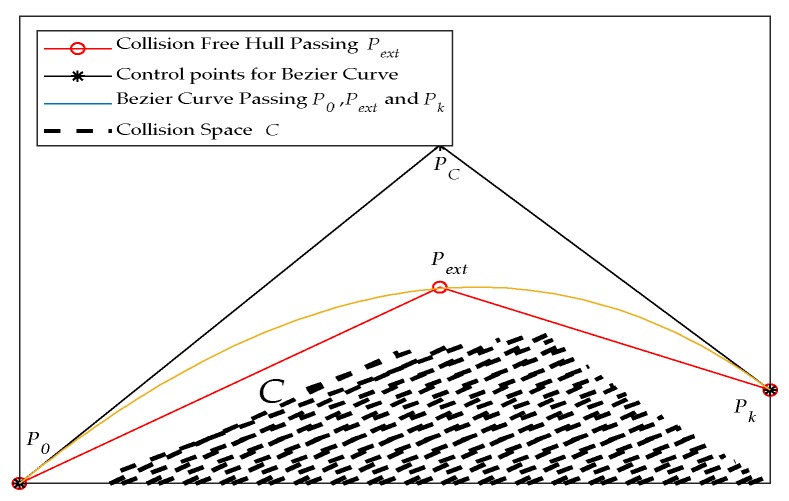
A Bezier curve passing *P*_0_, *P_ext_*, and *P_k_*, defined by control points *P*_0_, *P_C_*, and *P_k_*.

Given a path H, the designed sub-level path begins from $P_{0}$ and ends at $P_{k}$, where $\left|P_{0}P_{k}\right|=\alpha \cdot r$, $P_{k}\in H$, $\vec{P_{0}P_{k}}\cdot \vec{H}>0$. The purpose of the curve is to generate a path that avoids collision spaces C. It is easy to find an external point $P_{ext}$ such that the hull defined by $P_{0}P_{ext}$ and $P_{ext}P_{k}$ fully covers C. Taking Figure 9 as an example, to avoid collisions with C, a curve that connects $P_{0}P_{k}$ is formed. As long as $P_{0}P_{ext}$ and $P_{ext}P_{k}$ have no intersection point with C, a Bezier curve passing, $P_{0}$, $P_{ext}$, and $P_{k}$ forms a trajectory that meets the requirements. To form the curve, the absent control point $P_{C}$ can be calculated via

(3) $$P_{C}=P_{ext}-\frac{1}{2}\sqrt{\left|P_{0}-P_{ext}\right|\left|P_{k}-P_{ext}\right|}\left[\frac{P_{0}-P_{ext}}{\left|P_{0}-P_{ext}\right|}+\frac{P_{k}-P_{ext}}{\left|P_{k}-P_{ext}\right|}\right].$$

## 4. Experiment and Evaluation

### 4.1. Orientation Guidance Tests in Virtual Test-Fields

We designed a desktop testbed to observe participants’ path-following behavior in virtual test-fields. In the test, the designed orientation guidance wristband was used to provides tactile feedback to the person under test, and a flight control game joystick (PXN-2113 Pro) was used for the participant to provide real-time feedback regarding perceived orientations. Virtual paths were generated on test-fields in Matlab, while a virtual character was placed at the given position of the test-field. 

Four blindfolded volunteers (A, B, C, D) and four BVI volunteers (E, F, G, H) participated in the virtual field test. The participants all reported no irregularities with wrist and hand tactile perception. All of them were informed of the purpose of the test in advance, but none of them were aware of the details of the experiments before the tests. The research procedure and protocol obeyed the local human-related research ethics regulations.

**Figure 10:**
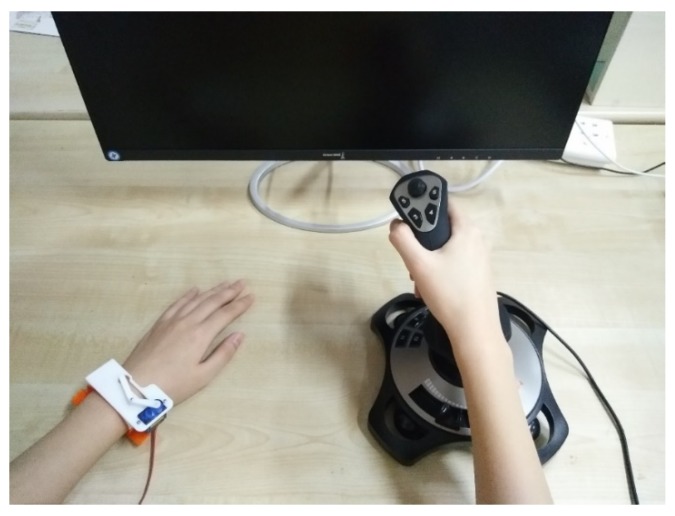
Virtual field test with the designed guidance wristband and feedback joystick.

In the test, a participant sat down in front of the testbed, wearing the proposed orientation guidance wristband on the left wrist. The joystick was held by the right hand, and the primary trigger button was under the control of the right index finger, as shown in Figure 10. The purpose of this set-up was to let the participant echo their perception regarding the orientation guidance introduced by the wristband. In the test, the real-time orientation guidance aiming to follow the pre-defined path was delivered to the participant via wristband, and the virtual character in the test field behaved as instructed by the joystick. A motion scaler that moderated the turning speed was arbitrarily set to 3%. 

**Figure 11:**
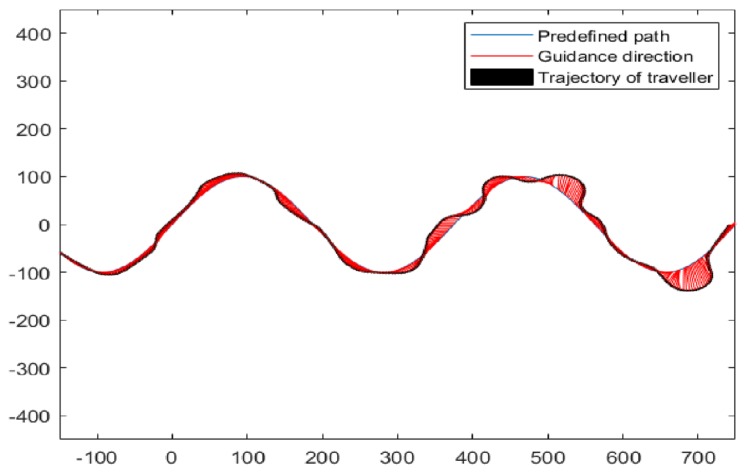
Predefined path, guiding force, and trajectory of traveler in virtual fields tests.

A standard sinusoidal path was applied as the predefined path, as shown in Figure 11. Two sets of tests (test α and β) were launched accordingly. In test α, participants received 15 min of training in virtual fields before entering the task. In test β, participants received training and practice in virtual fields until they felt confident in guidance. 

**Figure 12:**
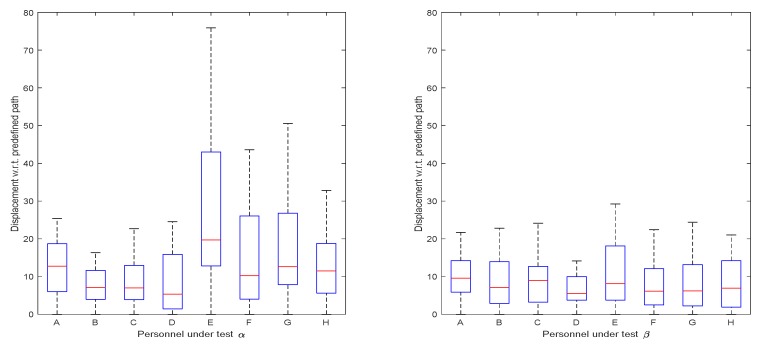
Displacement to predefined path in virtual field tests.

The displacements of participants to the predefined path were collected as shown in Figure 12. In test α, although the concept of following orientation guidance was simple, most participants were unable to steadily control the virtual character on the virtual test-fields by exclusively receiving orientation guidance from the designed wristband. One reason was that the controlled character always turned excessively, especially right after sharp turns. In the test, participant E reported that the sliding tactile feedback was difficult to perceive, causing a relatively larger displacement than other participants in test α. To deal with this, we adjusted the tightness of wristband. Thus, their displacement in test β was closer to the others. However, participant E reported an uncomfortable feeling after adjusting the tightness of the wristband. This phenomenon may have been caused by the diversity of each person’s skin tactile sensitivities. In general, the displacements in test β were smaller than those in test α, indicating that a series of training and practice sessions allows participants to rapidly adapt to the guidance tools and rules.

### 4.2. Orientation Guidance Tests in Real Test-Fields

**Figure 13:**
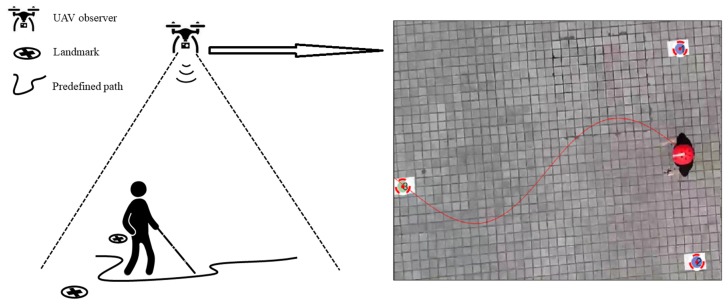
Field test scene. Left, an overlook mode unmanned aerial vehicle (UAV) is used to capture and transmit the positioning status of the participant and landmarks to the server to calculate the guiding direction. Right, the image from the UAV camera is shown, in addition to the auto-marked landmarks and the predefined path.

To evaluate the design in practice, we conducted field tests in obstacle-free real test-fields In the experiment, the Arduino-driven tactile wristband was mounted on the left wrist of the participant to provide orientation guidance, a Bluetooth module was used to receive real-time guidance messages, triple visual landmarks were used placed on the test ground for localization purposes, and an overlooking mode drone (SYMA XPRO 25) was used as an observer. As shown in Figure 13, the pre-defined sinusoidal path was uniquely determined by the triple visual landmarks, where the mid-point of the dual blue landmarks was set as the starting position, and the amplitude of the sinusoidal path was equal to half of the distance between dual blue landmarks. By matching the participant’s location with the path, the corresponding orientation guidance instruction was calculated and delivered to the wristband. There was no end point on the predefined path. Instead, the participant was guided to move along the sinusoidal path until 100 valid discrete data clips were collected. Test trials exceeding the boundary were regarded as failures and abandoned.

Note that the same eight participants in the virtual field tests took part in the field experiment. Three sets of tests (test γ, ∆, and θ) were launched. In test γ, participants received 15 min of training in a real test-field before the test. In test ∆, participants received training and practice in a real test-field until they felt confident. Test θ was conducted 24 h after test ∆, and the participants received training and practice in a virtual test-field until they felt confident. The five set of tests were conducted in the following sequence: α, γ, β, ∆, and θ.

**Figure 14:**
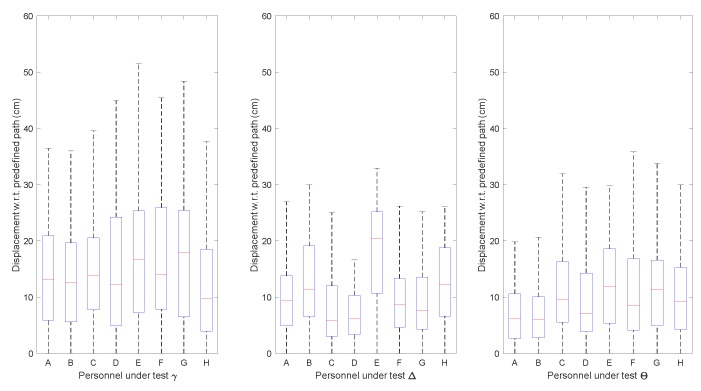
Displacement to predefined path in field tests.

The displacements of each participant in tests γ, ∆, and θ are shown in Figure 14. Even for the test without much training experience, the displacements were quite small. On one hand, this verified the efficiency of the wristband in orientation guidance in the real test-field. On the other hand, the participants in the real test-field were more careful. In the test, they often stopped or slowed down their movement for better orientation perception. Participant E still obtained the largest drift, implying that the personal skin tactile sensitivity features significantly influenced the adoption of the designed device. Apparently, after a short learning and practice period in real test-fields, the participants rapidly mastered the skill of following the guidance provided by the wristband. Test θ indicates that the training in virtual fields benefited participants using the wristband in real test-fields.

## 5. Discussion

The advances of machine intelligence and sensor technology significantly expanded the imagination space of modern scientific services and rehabilitation therapy for BVI people. Currently, the multimodal representation of the external world in the absence of a primary sensory capability requires not only a theoretical contribution but also human-centric design thinking solutions.

### 5.1. The Design Thinking in BVI Navigation Systems

BVI human–machine systems require more than task-fulfilling execution. Some navigation tasks easy for robots may be challenging for BVI users. On one hand, the ability to maintain a stable body reference in egocentric tasks is challenging for BVI users. On the other hand, the inherent mental status, involving the feeling of safety, pride, comfort, and fear, may affect distinct human cognition and behaviors. The design thinking double-diamond model is human-centered, whereby the needs of humans and objective factors are the main determinants of its design and delivery. Simple yet efficient, the design of the wristband provides continuous and reliable instructions in navigation, while distributing the cognitive load from hearing to tactile.

### 5.2. Motion Behavior Style Differences between subjects in Virtual Test-Field and Real Test-Field

By observing the tests on virtual test-fields and real test-fields, we realized that the real motion behaviors of BVI pedestrians were quite different from those in virtual field tests. Firstly, the subject in virtual fields always moved forward, while the frontal orientation changed over time. In contrast, the BVI pedestrians in real test-fields moved with more freedom, whereby they did not have to turn before moving or keep moving forward. In other words, the well-adapted game motion styles may not perfectly represent pedestrian motion styles in the real world. However, this kind of motion style may suit BVI semi-automatic driving perfectly, e.g., a BVI user driving a semi-automatic electric scooter.

### 5.3. Adaptive Tactile Stimulation and Reactions

The skin tactile sensing capability varied, especially for the wristband sliding tactile pressure. In the test, the subjects reported losing track of touch to varying degrees with the guidance wristband contactor. Fortunately, by gently pressing down the surface of the wristband, they were capable to retrack the pressure from the wristband contactor, since the contactor was pressed toward the wrist skin. However, this is not a long-term solution. Thus, a vibration supplement might be added to enhance the contactor sensation. Moreover, by monitoring, recording, and learning from individual specifications regarding skin sensibility, a customized tactile stimulation mechanism would be helpful for assisting BVI users in travel. For example, users with more wrist fat tend to receive stronger tactile stimulation. 

### 5.4. Orientation Finding and Spatial Perception Rehabilitation 

The inner clock orientation exercise is the most popular orientation finding training method for BVI people. The proposed design of the wristband borrowed insight from spatial perception training, and it was developed to help enhance the capability of orientation finding in locomotion. In the experiments, we found that the blindfolded participants performed better than the BVI participants in virtual test-fields. One of the reasons is that the blind-folded participants had a similar experience when controlling virtual characters in video games. However, when it came to the real test-field, the performances of the blindfolded and BVI participants showed inconspicuous differences. The extensive training in virtual fields also benefited the experience in real test-fields, which implies that testing in a virtual scenario may potentially be a usefully tool in spatial perception rehabilitation. 

## 6. Conclusions

In this work, we presented a novel orientation guidance design for a BVI navigation system. The design followed the double-diamond design model and achieved human-centric innovation while ensuring technical feasibility and economic viability. It provided continuous and smooth instructions via a sliding tactile sensor, to overcome the numerous limitations of existing acoustic and vibration orientation guidance solutions. We also presented a Bezier curve-based path-following algorithm to support the orientation guidance. To verify the efficiency, we prototyped and tested the design in both virtual test-fields and real test-fields. The results confirmed the efficiency of the design, and also implied that extensive training in a virtual test-field also benefits users in real test-fields. The testbed of the virtual field test may potentially be useful in the spatial perception rehabilitation of BVI people.

The prototyped design has an approximate weight of 50 g and a cost of about $2 United States dollars (USD) plus the wireless communication module. Such a lightweight and commercially affordable design has the potential to become a ubiquitous guiding accessory for general BVI smart navigation systems.

In future work, the restriction of the weak tactile stimulation will be resolved without negatively influencing the usability and user experience. Moreover, a comprehensive experimental user statistical study in a larger area and in a crowded urban area will be conducted to evaluate the capability of the orientation guidance tool and its impact on BVI people’s daily life and behavior.
